# An electronic trigger tool to optimise intravenous to oral antibiotic switch: a controlled, interrupted time series study

**DOI:** 10.1186/s13756-017-0239-3

**Published:** 2017-08-15

**Authors:** Marvin A. H. Berrevoets, Johannes (Hans) L. W. Pot, Anne E. Houterman, Anton (Ton) S. M. Dofferhoff, Marrigje H. Nabuurs-Franssen, Hanneke W. H. A. Fleuren, Bart-Jan Kullberg, Jeroen A. Schouten, Tom Sprong

**Affiliations:** 1 Department of Internal Medicine and Infectious Diseases, Radboudumc, Nijmegen, the Netherlands; 2 Department of Clinical Pharmacy, Canisius-Wilhelmina Hospital, Nijmegen, the Netherlands; 3 Department of Internal Medicine, Canisius-Wilhelmina Hospital, Nijmegen, the Netherlands; 4 Department of Medical Microbiology and Infectious Diseases, Canisius-Wilhelmina Hospital, Nijmegen, the Netherlands; 5 Department of Intensive Care, Canisius-Wilhelmina Hospital, Nijmegen, the Netherlands

**Keywords:** Decision support system, Antimicrobial stewardship, Iv-oral switch, Quality of care

## Abstract

**Background:**

Timely switch from intravenous (iv) antibiotics to oral therapy is a key component of antimicrobial stewardship programs in order to improve patient safety, promote early discharge and reduce costs. We have introduced a time-efficient and easily implementable intervention that relies on a computerized trigger tool, which identifies patients who are candidates for an iv to oral antibiotic switch.

**Methods:**

The intervention was introduced on all internal medicine wards in a teaching hospital. Patients were automatically identified by an electronic trigger tool when parenteral antibiotics were used for >48 h and clinical or pharmacological data did not preclude switch therapy. A weekly educational session was introduced to alert the physicians on the intervention wards. The intervention wards were compared with control wards, which included all other hospital wards. An interrupted time-series analysis was performed to compare the pre-intervention period with the post-intervention period using ‘% of i.v. prescriptions >72 h’ and ‘median duration of iv therapy per prescription’ as outcomes. We performed a detailed prospective evaluation on a subset of 244 prescriptions to evaluate the efficacy and appropriateness of the intervention.

**Results:**

The number of intravenous prescriptions longer than 72 h was reduced by 19% in the intervention group (*n* = 1519) (*p* < 0.01) and the median duration of iv antibiotics was reduced with 0.8 days (*p* = <0.05). Compared to the control group (*n* = 4366) the intervention was responsible for an additional decrease of 13% (*p* < 0.05) in prolonged prescriptions.

The detailed prospective evaluation of a subgroup of patients showed that adherence to the electronic reminder was 72%.

**Conclusions:**

An electronic trigger tool combined with a weekly educational session was effective in reducing the duration of intravenous antimicrobial therapy.

**Electronic supplementary material:**

The online version of this article (doi:10.1186/s13756-017-0239-3) contains supplementary material, which is available to authorized users.

## Background

Most patients with an infection that requires inpatient treatment initially receive empirical intravenous (iv) antimicrobial therapy. When the patient clinically improves within 48 h and results from microbiology cultures and other tests become available these iv antibiotics may be switched to oral therapy, with the exception of certain clinical conditions that necessitate prolonged iv treatment (e.g. *Staphylococcus aureus* bacteremia, endocarditis, meningitis). Previous studies have shown that a timely switch from intravenous to oral therapy is safe and reduces risk of complications related to intravenous treatment, healthcare costs and duration of hospitalization [1–6].

A Dutch study found that for patients with community-acquired pneumonia, a switch to oral antibiotics was possible in 46% of the patients on day 3 of treatment, but was not performed in 40% of eligible switch opportunities [7]. Barriers that preclude switching include misconceptions, practical considerations and organizational factors [7, 8]. Different approaches have been deployed to incorporate switch therapy into daily practice. However, the major conversion programmes evaluated in the literature were labour intensive interventions [3, 4] and not aimed at solving the barriers to timely switching therapy from iv to oral [5]. Previous research showed that introducing switch therapy into daily practice by a computerized trigger tool was effective in promoting iv to oral switch therapy [9, 10]. However, these reminders were produced with low specificity, were only performed for a limited number of antibiotics or had a low adherence rate.

The goal of this study was to evaluate the effect of a combined intervention targeting different barriers that preclude switching. The first intervention, a computerized reminder, was chosen to target both practical and organisational factors, while continuous education and feedback on iv-oral switch practice and promoting the use of a so called ‘switch card’ aimed to improve misconceptions about switching. The computerized reminder relies on an electronic trigger tool, which identifies patients who are candidates for antibiotic switch therapy.

With this combined intervention we aimed to improve the rate of safe iv to oral antibiotic switch on internal medicine wards.

## Methods

This controlled intervention study was performed at the Canisius-Wilhemina Hospital, Nijmegen, the Netherlands, a 455-bed non-academic teaching hospital. The study was performed during a 26-month period. The pre-intervention period was defined as the first 13 months of the study period and the post-intervention period was defined as the last 13 months of the study period. The intervention was carried out at the internal medicine wards. The control wards included all other hospital wards.

### Intervention

#### Computerised reminders

The multidisciplinary Antimicrobial Stewardship team (AST) (comprised of an infectious disease specialist, microbiologist and clinical pharmacist) created an automatic warning system that identifies candidates who are eligible for iv-oral switch therapy. The tool was based on the Dutch National Antibiotic Switch guidelines [3] and was developed in Crystal Reports® using data from the hospital pharmacy database and the clinical chemistry department.

Patients were identified as eligible for iv-oral switch therapy when antibiotic treatment had been prescribed 48 to 72 h previously. To alert the physician of the possibility of iv-oral switch an automated reminder was sent on day 3 of the treatment. This trigger tool automatically checked whether the following clinical or pharmacological conditions precluded switch therapy: (i) increase in CRP during iv treatment; (ii) neutrophils <0,5*10^9^/ml; (iii) leukocytes <1*10^9^/ml; (iv) usage of parenteral medication only or total parenteral nutrition, as an inability for oral intake predictor; (v) specific or high dose antibiotic suggesting severe infection (e.g. endocarditis, meningitis) (Additional file 1: Appendix 1). Patients meeting one or more of these criteria were not selected for switch therapy. If a single patient received more than one antibiotic, each single prescription (given for more than 48 h) was checked by the trigger tool to determine eligibility for iv-oral switch. Every morning, for each eligible patient, a written notice was automatically generated and sent out to the department secretary. This form contained identifying data of the patient, the antibiotic(s) that were eligible to be switched, and possible oral options or alternatives for these antibiotics. The form was presented to the attending physician during ward rounds by the department secretary. The decision whether to switch or not was made by the physician, based on the clinical situation of the patient. For each patient the physician was asked to fill in a form on which considerations concerning the switch were listed (ability to switch, impeding reasons). Reasons precluding iv-oral switch were categorized as ‘clinical instability’, no oral intake possible, severe infection (e.g. *Staphylococcus aureus* bacteremia, meningitis, endocarditis) or resistant micro-organisms for which no oral therapy is available.

A second reminder was sent when the iv medication order was started 96 to 120 h before and no oral switch had yet been performed, based on the same algorithm as mentioned earlier. To prevent alert fatigue a maximum of two reminders per medication order per patient was chosen.

Due to organizational factors no switch forms were distributed during the weekend.

#### Educational program

The start of the intervention was preceded by an educational program. Pocket cards with the switch protocol were presented to each physician. The hospital protocol (Additional file 2: Appendix 2) recommends to choose an oral antibiotic based on culture results. When no oral formulation of the iv antibiotic eligible for switch is available and culture results are negative, an oral antibiotic covering a similar spectrum as the empirically started iv antibiotic is recommended (e.g. ceftriaxon should be switched to oral amoxicillin/clavulanic acid).

To optimize adherence, direct feedback and education was given to the physicians working on the intervention wards regarding the returned switch forms in a weekly short meeting.

During this 15-min meeting (called “Switch of the week”) one or more of the completed switch forms were selected by an infectious disease specialist (T.S.) and the content was presented to the physicians. Both appropriate and inappropriate decisions of the physicians were discussed and feedback was given on the decisions made.

### Prospective observational study to assess the effectiveness of the intervention

To determine the effectiveness of the intervention in promoting iv-oral switch therapy, data of all iv antibiotic prescriptions was collected from the hospital pharmacy database for the whole study period. The intervention group was represented by patients from the internal medicine wards, while the control group was represented by patients in the same hospital during the same period from all other wards. The intervention was carried out at the internal medicine wards during the last 13 months of the study period. The percentage of intravenous prescriptions that exceed 72 h (% > 72 h) and median treatment duration in days of intravenous therapy on the intervention wards were calculated and compared to the control period and the control wards.

### Detailed prospective evaluation of a subset of prescriptions to assess appropriateness

During the first 4 months of the intervention period, antibiotic prescriptions selected by the trigger tool were included in a detailed analysis to evaluate the efficacy and appropriateness of the intervention. The following patient data were collected to categorize the switch: patient’s age and gender, source of infection, time of hospitalization, antimicrobial treatment, starting date, dose, body temperature, blood leukocyte count, blood neutrophil count, culture results and reasons which impeded the switch, based on the returned switch-forms. The appropriateness of the iv to oral switch was evaluated by an infectious disease specialist (TS). Options to categorize the conversion were: ‘appropriate switch’, ‘inappropriate switch’, ‘appropriate continuation of iv therapy’ and ‘inappropriate continuation of iv therapy. Categorization was based on the following switch criteria: no indication for prolonged iv antibiotic treatment (e.g. *Staphylococcus aureus* bacteremia, endocarditis, meningitis), improving vital signs, susceptible micro-organism (if cultured) for oral antibiotics, and the presence of a functional tractus digestivus. A patient who fulfilled these 4 switch criteria and who was switched to oral antibiotic therapy was categorized as an appropriate switch (Additional file 2: Appendix 2 – Table S3).

#### Endpoints

The primary endpoints were the change in percentage of iv treatment exceeding >72 h for all patients in both the intervention group and control group between the pre- and post-intervention period and the percentage of patients appropriately switched to oral therapy on day 3 of treatment. Secondary outcome was the change in median treatment duration.

#### Statistics

All data was analyzed using the IBM SPSS20 software package. We performed an interrupted time series analysis (ITSA), for which we used segmented linear regression to assess the significance of changes in level and slope of the regression lines before and after the introduction of the intervention for both the intervention and control groups [11, 12]. This methodology evaluates data collected at multiple time points before and after an intervention to detect whether the intervention had a greater effect than the expected secular trend. An abrupt intervention effect constitutes a change in the level of the outcome directly after the intervention is implemented. The slope represents a gradual change in the outcome parameter during the segment [12]. We divided the dataset into monthly periods (of which there were 26; 13 periods before the intervention started and 13 periods after the intervention started). The analysis was performed for the outcomes percentage of iv treatment >72 h, and median treatment duration in both the intervention and control group. We used a *p*-value of <0.05 as a threshold for all statistical tests.

Adherence to the intervention was determined by dividing the prescriptions that were appropriately switched (numerator) by the total amount of prescriptions eligible for switch (denominator).

#### Ethics statement

As iv to oral antibiotic switch therapy is proven to be safe and effective and is an essential element of many antimicrobial stewardship programs we considered timely switch as a standard of care, and our study is an evaluation of a new method to promote this standard of care. Patient data were collected anonymously. This study does not fall within the remit of the Medical Research involving Human Subjects Act (WMO). Therefore the study can be carried out in the Netherlands without an approval by an accredited research ethics committee and without explicit informed consent of the participants.

## Results

### Baseline characteristics

During the whole study period, 1519 patients on the intervention wards and 4366 patients on the control wards received intravenous antibiotics (Table 1). At baseline, on the intervention wards 50.5% of prescriptions were given for >72 h versus 49.8% on the control wards.

**Table 1:** Baseline characteristics of patients on the intervention wards and controls wards during the pre-intervention and post-intervention periods

Characteristic	Intervention wards		Control wards	
	Pre-intervention period	Post-intervention period	Pre-intervention period	Post-intervention period
Patients	771	748	2154	2212
Hospital admissions	880	835	2458	2478
Medication orders (iv antibiotics)	1781	1534	4769	4294
Mean age [range]	68.3 [16–101]	68.4 [17–101]	54.8 [1–100]	54.1 [0–98]
Female (%)	405 (53%)	371 (50%)	1029 (48%)	1073 (49%)

#### Prospective observational study to assess the effectiveness of the intervention

**Fig. 1:**
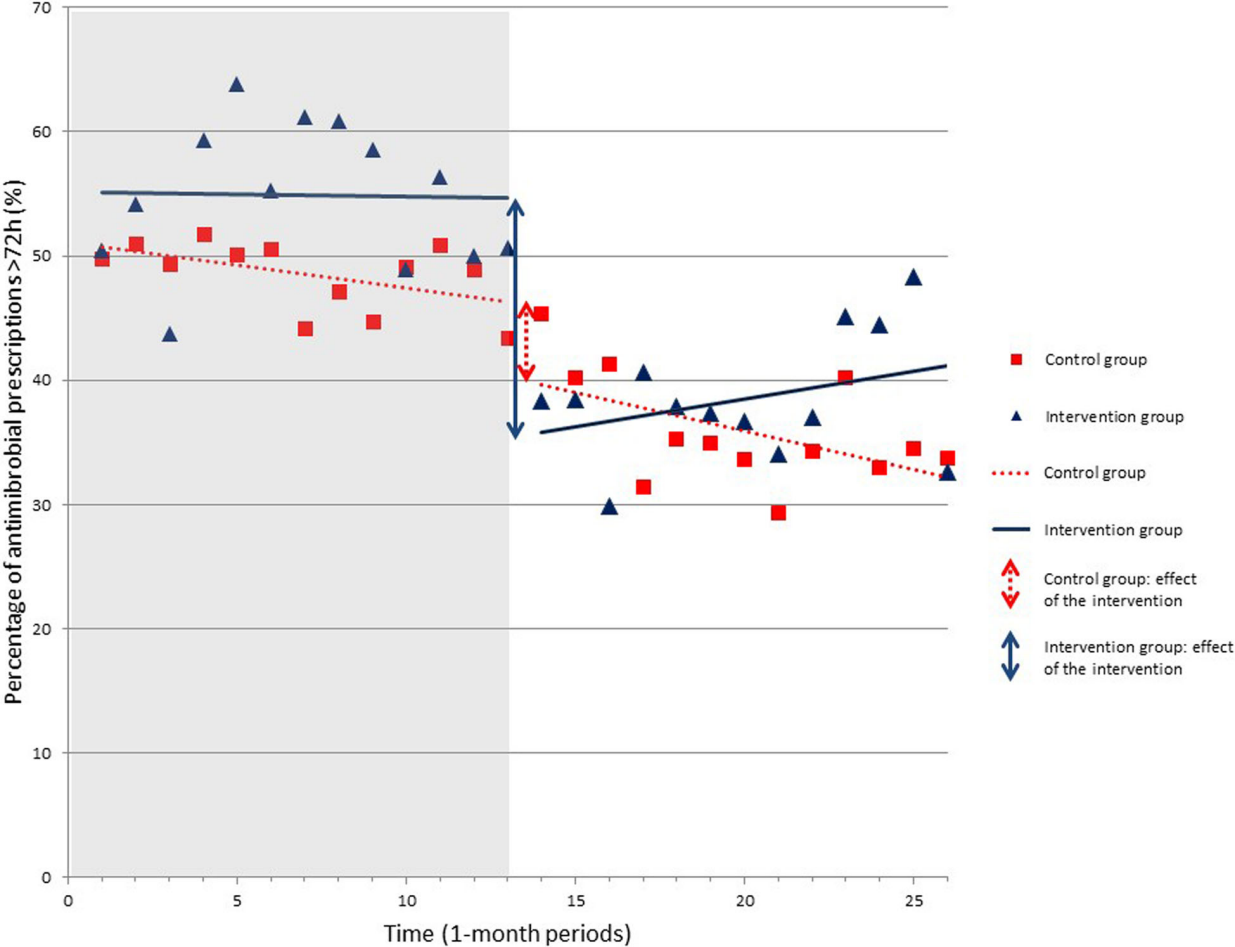
Interrupted time series analysis of percentage of iv antimicrobial prescriptions >72 h. The grey area in the chart represents the pre-intervention period, the white area the post-intervention period. The squares represent the control group, where the triangles represent the intervention group. A trend line has been drawn through the data points for each group during the pre- and post-intervention period. The time series analysis demonstrated a significant decrease in the percentage of antimicrobial prescriptions in the intervention group (solid lines and solid double arrow, reduction 19.3%; *p* < 0.01) and in the control group (dashed lines and dashed double arrow, reduction 6.1%; *p* < 0.05), with an additional reduction of 13.2% (*p* < 0.05) in favour of the intervention group

Using interrupted time-series analysis, we compared the percentage of iv antibiotic prescriptions >72 h in the pre-intervention and post-intervention periods. There was a significant decrease (reduction 19.3%, *p* < 0.001; Table 2, Fig. 1) in the intervention group. We observed a significant but smaller decrease in the control group as well (reduction 6.1%, *p* < 0.05; Table 2, Fig. 1). The difference between the intervention and control group showed a significant additional reduction of intravenous antibiotic usage of 13.2% (*p* = 0.014) in favour of the intervention group.

**Table 2:** Change in percentage of iv antibiotic prescriptions >72 h and median antibiotic duration (days) during the pre-intervention and post-intervention period and comparison between the intervention and control group using interrupted time series analysis

	Slope during the pre-intervention period (SE)	*P*	Change in level after intervention^a^ (SE)	*P*	Slope during the post-intervention period^b^ (SE)	*P*
Percentage of iv antibiotic prescriptions >72 h (%)						
Intervention group	−0.03 (0.42)	0.94	−19.30 (4.41)	<0.01	0.48 (0.59)	0.43
Control group	−0.36 (0.25)	0.16	−6.12 (2.61)	<0.05	−0.26 (0.35)	0.46
Difference between intervention and control group	0.33 (0.48)	0.50	−13.17 (5.13)	<0.05	0.74 (0.68)	0.29
Median duration of iv antibiotics (days)						
Intervention group	−0.02 (0.03)	0.49	−0.77 (0.29)	0.015	0.02 (0.04)	0.63
Control group	−0.38 (0.03)	0.16	−0.15 (0.28)	0.59	0.04 (0.04)	0.32
Difference between intervention and control group	0.02 (0.04)	0.62	0.62 (0.41)	0.14	−0.02 (0.05)	0.72

^a^Measures the immediate impact of the intervention

^b^Measures the long-term impact over time of the intervention

During the post-intervention period, a non-significant increase in % of prescriptions >72 h was observed in the intervention-group (solid line in the white area, slope of +0.48% / month, *p* = 0.43; Fig. 1) and a non-significant decrease in the control group (dashed line in the white area, slope of −0.26% /month, *p* = 0.46; Fig. 1), with no significant difference between these groups.

Median duration of antibiotic usage on the intervention wards was decreased by 0.8 day (4.0 to 3.2, *p* = 0.015) (Table 2) after the intervention was introduced. There was no significant decrease observed on the control wards or between the intervention and control wards.

### Detailed prospective evaluation of a subset of prescriptions to assess appropriateness

**Fig. 2:**
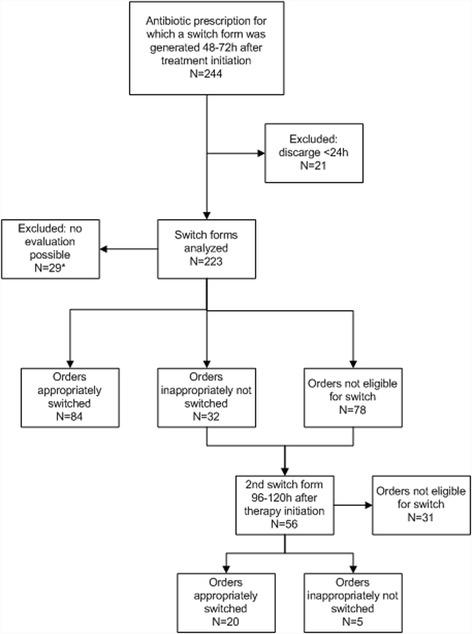
Prospective evaluation of a subset of prescriptions. *no switch form returned or switch form generated during weekend days

During a 4-month period there were 244 unique antibiotic medication orders for which the intervention was activated, 21 prescriptions were excluded, since these patients left the hospital on the day of the possible iv-oral switch. This resulted in 223 medication orders for a detailed analysis (Fig. 2).

Of all medication orders, 116 were eligible for switch. Of these, 84 were switched correctly (72%) and 32 (28%) were incorrectly not switched. A total of 78 orders were not eligible for switch. For 29 orders there was no evaluation possible, either because the switch order was generated in the weekend or there was no form filled in. Most of the patients with medication orders that were candidates for conversion had pneumonia (24.7%). The mean age was 71 years and most patients were female (53%). Table 3 shows data about the infections treated, and antibiotics prescribed during this phase of the study. The antibiotic agent that was most often eligible for switch was cefotaxime (27.5%).

**Table 3:** Detailed prospective analysis; indications and antibiotics prescribed for 244 analysed switch forms

Indication		Antibiotic	
Pneumonia	24.7%	cefotaxim	27.5%
Sepsis	20.4%	ciprofloxacin	19.0%
Pyelonephritis/urosepsis	18.6%	amoxicillin	16.2%
Abdominal infection	17.6%	metronidazole	15.0%
Skin and soft tissue infection	11.5%	cefazolin	12.1%
Unknown indication	2.5%	penicillin	4.5%
Gastro-enteritis	2.5%	clindamycin	4.0%
Bone and joint infection	1.1%	fluconazole	0.8%
Other	1.1%	ceftriaxone	0.4%
		flucloxacillin	0.4%

During the detailed prospective evaluation*,* the digital switch forms were generated at two points in time. In case the first form did not result in a switch, a second form was generated from 96 to 120 h after therapy initiation. This resulted in 56 switch forms generated of which 20 lead to a correct switch and no incorrect switches, 31 were not eligible for iv-oral switch and 5 were indicated as incorrectly not switched (Fig. 2).

This detailed analysis showed that no patients were inappropriately switched.

## Discussion

A computerized intervention in combination with an educational and feedback program, safely reduces the percentage of prolonged intravenous antibiotic use in hospital wards with 19%.

Many studies have demonstrated that iv-oral switch therapy is associated with a reduced length of iv therapy and low clinical failure rate [6]. In most studies an investigator identified cases eligible for switch by manual data extraction. This is time consuming and has limited feasibility in daily practice.

In this study, a simple automated trigger tool in our clinical pharmacy database, selected patients eligible for switch. An automated reminder was printed and sent to the ward prompting the physician to perform an iv-oral switch.

By automating the intervention, we were able to systematically reduce workload, which was a problem in many other studies [13]. In addition, we gave direct feedback to the participating physicians improving adherence to the national iv-oral switch guideline. This resulted in a high switch-rate of 72%.

The use of a computerized intervention has been investigated in a limited number of studies. Fisher et al. developed computerized interventions which automatically sent reminders to the physician, when an iv-oral switch was possible. Of the iv orders 21.6% were replaced by an oral agent, and 14% of the selected orders were discontinued [10]. However, this intervention was initiated on only 5 different categorized target drugs and adherence was low. Another study showed a decrease of iv use of levofloxacin and ciprofloxacin when displaying electronic alerts [9].

Beeler et al. [14] performed a prospective, controlled trial using an electronic reminder to stimulate iv-oral switch. Their study resulted in a decrease of total iv duration of 17.5% in the intervention group, and a switch-rate of 26.6%. Unfortunately, they did not report on the amount of patients who were not eligible for switch, which could explain the difference with the adherence rate in our own study.

Our study has several strengths. First, we used a large patient population and physician response to the switch reminder was high (72%). The advantage of our switch intervention lies in the possibilities of automatically identifying patients eligible for switch and alerting the physician. Besides the distribution of the switch-forms by the department secretary, no man-power is required for the continuous operation of the trigger tool, furthermore the educational program only takes 15-min a week. This enables antimicrobial stewardship teams to focus on other stewardship activities. The implementation of more sophisticated electronic health record systems (EHRS), which provide electronic alerts, could contribute to an additional reduction of workload [14, 15].

A recent survey conducted in France showed that human resources needed to implement AST activities were estimated at 3.6 full-time equivalent (FTE) positions/1000 acute care beds for antibiotic/infectious disease lead supervisors, at 2.5 FTE/1000 beds for pharmacists, and at 0.6 FTE/1000 beds for microbiologists [16]. Most hospitals in the Netherlands have restricted manpower for AST and can only focus on a limited number of stewardship activities. Our intervention is free and effective and can contribute to a simple and successful implementation of an iv-oral switch program.

Second, our controlled, interrupted time series analysis design is more robust than (un)controlled before and after analyses or uncontrolled interrupted time series designs used in most stewardship studies [17]. We were able to identify a significant difference in intravenous usage of antibiotics >72 h on the intervention wards (reduction 19%). However, in the control wards a significant effect (reduction 6%) was also found. This effect could possibly be contributed to the nationwide and local hospital attention for antimicrobial stewardship during the time of the study. Nevertheless, our iv-oral switch intervention was responsible for an additional effect of 13% reduction in iv antibiotic usage >72 h in favor of the intervention wards.

Our study has some limitations. First, baseline characteristics of the intervention and control groups were not comparable. The intervention was implemented at internal medicine wards and the control wards represented all other hospital wards; the baseline percentage of intravenous antibiotic use for >72 h was higher (+0.7%) in the intervention group. Therefore, the effect of our intervention may not be transferable to all hospital wards. Second, our intervention was not fully effective, from all antibiotics eligible for switch, 28% were incorrectly not switched and due to organizational factors we were not able to implement the intervention during the weekend. This indicates that there is still ample room for improvement. Since physicians were asked to fill in reasons for not switching, these will be used to improve the educational program. Third, patients who received solely iv antibiotics could have been incorrectly excluded, while they may have been candidates for iv-oral switch therapy. Nowadays, with modern EHRS, more sophisticated algorithms that are based on general accepted switch criteria, and that incorporate both clinical, pharmaceutical and laboratory details could be developed.

Finally, this was a single center study, therefore, results may not be widely generalizable. However, the iv-oral switch algorithm was designed based upon commonly used clinical and laboratory criteria. With the help of an information technician the implementation in other hospitals with an electronic prescribing system should be possible.

## Conclusion

This study showed the efficacy of an electronic trigger tool combined with targeted education on iv-oral switch. The intervention significantly decreased the amount of intravenously administered antibiotics used for >72 h by 19%.

This trigger tool enables hospital ASTs to implement a feasible and effective stand-alone iv-oral switch strategy, winning them valuable time to address more complicated issues of antimicrobial prescribing in daily practice.

## Additional files

Additional file 1: Appendix 1. Electronic trigger tool algorithm to identify patients eligible for iv to oral switch. ^#^high dose of a penicillin or cephalosporin only indicated for severe infection (e.g. endocarditis or meningitis) or an antibiotic class which is given only if no oral formulation is available (carbapenem). (JPEG 63 kb)

Additional file 2: Appendix 2. Protocol for iv to oral switch therapy. (DOCX 17 kb)

## Abbreviations

AST: Antimicrobial stewardship teams
EHRS: Electronic health record systems
FTE: Full-time equivalent
ITSA: Interrupted time series analysis
IV: Intravenous
