# Supplementary figures and images for: An electronic trigger tool to optimise intravenous to oral antibiotic switch: a controlled, interrupted time series study
Source: Antimicrob Resist Infect Control. 2017 Aug 15;6:81. doi: 10.1186/s13756-017-0239-3 (PMC5558766; doi:10.1186/s13756-017-0239-3)

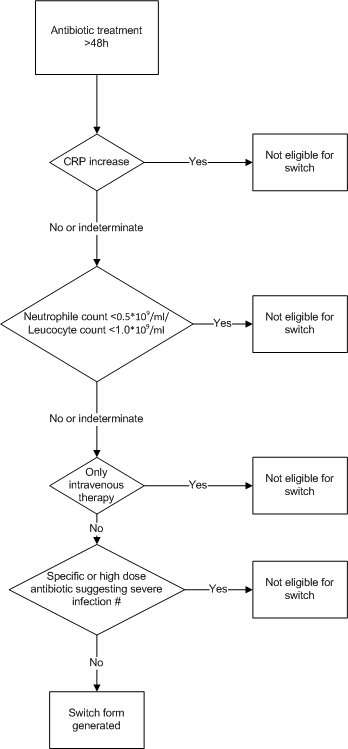

Supplement: Supplementary file 1 — Electronic trigger tool algorithm to identify patients eligible for iv to oral switch. #high dose of a penicillin or cephalosporin only indicated for severe infection (e.g. endocarditis or meningitis) or an antibiotic class which is given only if no oral formulation is available (carbapenem). (JPEG 63 kb) [file 13756_2017_239_MOESM1_ESM.jpg]
